# The Anticoagulant Nafamostat Potently Inhibits SARS-CoV-2 S Protein-Mediated Fusion in a Cell Fusion Assay System and Viral Infection In Vitro in a Cell-Type-Dependent Manner

**DOI:** 10.3390/v12060629

**Published:** 2020-06-10

**Authors:** Mizuki Yamamoto, Maki Kiso, Yuko Sakai-Tagawa, Kiyoko Iwatsuki-Horimoto, Masaki Imai, Makoto Takeda, Noriko Kinoshita, Norio Ohmagari, Jin Gohda, Kentaro Semba, Zene Matsuda, Yasushi Kawaguchi, Yoshihiro Kawaoka, Jun-ichiro Inoue

**Affiliations:** 1 Research Center for Asian Infectious Diseases, Institute of Medical Science, University of Tokyo, Tokyo 108-8639, Japan; mizuyama@ims.u-tokyo.ac.jp (M.Y.); jgohda@ims.u-tokyo.ac.jp (J.G.); zmatsuda@ims.u-tokyo.ac.jp (Z.M.); ykawagu@ims.u-tokyo.ac.jp (Y.K.); 2 Division of Virology, Department of Microbiology and Immunology, Institute of Medical Science, University of Tokyo, Tokyo 108-8639, Japan; kisomaki@ims.u-tokyo.ac.jp (M.K.); ytsakai@ims.u-tokyo.ac.jp (Y.S.-T.); kenken@ims.u-tokyo.ac.jp (K.I.-H.); mimai@ims.u-tokyo.ac.jp (M.I.); yoshihiro.kawaoka@wisc.edu (Y.K.); 3 Department of Virology 3, National Institute of Infectious Diseases, Musashimurayama, Tokyo 208-0011, Japan; mtakeda@nih.go.jp; 4 Disease Control and Prevention Center, National Center for Global Health and Medicine, Tokyo 162-8655, Japan; nkinoshita@hosp.ncgm.go.jp (N.K.); nohmagari@hosp.ncgm.go.jp (N.O.); 5 Department of Life Science and Medical Bio-Science, Waseda University, Shinjuku-ku, Tokyo 162-8480, Japan; ksemba@waseda.jp; 6 Division of Molecular Virology, Department of Microbiology and Immunology, The Institute of Medical Science, The University of Tokyo, Tokyo 108-8639, Japan; 7 Influenza Research Institute, Department of Pathobiological Sciences, School of Veterinary Medicine, University of Wisconsin-Madison, Madison, WI 53711, USA; 8 Department of Special Pathogens, International Research Center for Infectious Diseases, Institute of Medical Science, University of Tokyo, Tokyo 108-8639, Japan; 9 Senior Professor Office, University of Tokyo, Tokyo 113-0033, Japan

**Keywords:** SARS-CoV-2, TMPRSS2, fusion inhibitor

## Abstract

Although infection by SARS-CoV-2, the causative agent of coronavirus pneumonia disease (COVID-19), is spreading rapidly worldwide, no drug has been shown to be sufficiently effective for treating COVID-19. We previously found that nafamostat mesylate, an existing drug used for disseminated intravascular coagulation (DIC), effectively blocked Middle East respiratory syndrome coronavirus (MERS-CoV) S protein-mediated cell fusion by targeting transmembrane serine protease 2 (TMPRSS2), and inhibited MERS-CoV infection of human lung epithelium-derived Calu-3 cells. Here we established a quantitative fusion assay dependent on severe acute respiratory syndrome coronavirus 2 (SARS-CoV-2) S protein, angiotensin I converting enzyme 2 (ACE2) and TMPRSS2, and found that nafamostat mesylate potently inhibited the fusion while camostat mesylate was about 10-fold less active. Furthermore, nafamostat mesylate blocked SARS-CoV-2 infection of Calu-3 cells with an effective concentration (EC)_50_ around 10 nM, which is below its average blood concentration after intravenous administration through continuous infusion. On the other hand, a significantly higher dose (EC_50_ around 30 μM) was required for VeroE6/TMPRSS2 cells, where the TMPRSS2-independent but cathepsin-dependent endosomal infection pathway likely predominates. Together, our study shows that nafamostat mesylate potently inhibits SARS-CoV-2 S protein-mediated fusion in a cell fusion assay system and also inhibits SARS-CoV-2 infection in vitro in a cell-type-dependent manner. These findings, together with accumulated clinical data regarding nafamostat’s safety, make it a likely candidate drug to treat COVID-19.

## 1. Introduction

Infection by severe acute respiratory syndrome coronavirus 2 (SARS-CoV-2), the causative agent of coronavirus pneumonia disease (COVID-19), is spreading rapidly worldwide [1]. As yet, no drug has been shown to be sufficiently effective for treating COVID-19. Therefore, drug repurposing offers potentially the quickest path toward disease treatment.

The genomic RNA of coronaviruses is surrounded by an envelope [2]. Initiation of viral entry requires two steps. In the first step, the Spike (S) protein in the viral envelope, binds to its receptor present in the plasma membrane through its receptor-binding domain (RBD), after S protein is cleaved into S1 and S2 proteins by some cellular proteases. SARS-CoV and SARS-CoV-2 use angiotensin converting enzyme 2 (ACE2), while the Middle East respiratory syndrome coronavirus (MERS-CoV) uses CD26 as a receptor. Secondly, S2 protein is cleaved by either cell surface transmembrane serine protease 2 (TMPRSS2) or lysosomal protease cathepsins. This cleavage is called priming, and exposes the fusion peptide in S2 protein, allowing it to attach to the plasma or endosomal membrane, resulting in the fusion between the viral envelope and the cellular membrane (envelope fusion). This fusion allows the viral RNA to enter the cytoplasm, where it replicates. It has been recently demonstrated that a proprotein convertase (PPC) motif present at the S1/S2 boundary of SARS-CoV-2 S protein is cleaved by the protease furin, s step crucial for efficient viral entry that probably acts by enhancing the interaction of RBD with ACE2 [3]. Since the furin-catalyzed pre-activation of S protein was not observed in SARS-CoV, it could be involved in COVID-19-unique disease development [3]. While furin is ubiquitously expressed [4], recent analysis of single-cell RNA-seq datasets from human tissues revealed that TMPRSS2 is expressed in a cell type specific manner [5,6]. Therefore, SARS-CoV-2 likely enters cells lacking TMPRSS2 through the cathepsin-dependent endosome pathway. Nevertheless, TMPRSS2-knockout resulted in reduced spread of SARS-CoV and MERS-CoV in the airways accompanied by reduced severity of lung pathology in a mouse model [7]. Therefore, furin and TMPRSS2 are likely crucial for SARS-CoV-2 spread and disease development in vivo, and targeting them either by inhibiting their catalytic activity or suppressing their expression is likely to be an effective strategy to cure COVID-19.

We have previously reported that nafamostat mesylate, an existing Japanese drug used for acute pancreatitis and disseminated intravascular coagulation (DIC), effectively inhibits MERS-CoV S protein-mediated membrane fusion by targeting TMPRSS2 priming activity [8]. We did this using the cell fusion assay monitored by the dual split protein (DSP) reporter to screen the FDA-approved drug library. Nafamostat mesylate potently inhibited MERS-CoV infection of lung epithelium-derived Calu-3 cells.

In this study, we established an experimental assay system monitoring ACE2- and TMPRSS2-dependent SARS-CoV-2 S protein-mediated membrane fusion, in 293FT and Calu-3 cells and found that nafamostat mesylate potently blocked SARS-CoV-2 S protein-mediated fusion in a cell fusion assay system and viral infection in vitro in a cell-type-dependent manner.

## 2. Materials and Methods

### 2.1. Protease Inhibitors and Anti-Coagulants

Nafamostat mesylate (Tokyo Chemical Industry, Tokyo, Japan), camostat mesylate (Wako, Tokyo, Japan), gabexate mesylate (Tokyo Chemical Industry, Tokyo, Japan), edoxaban, apixaban, rivaroxaban, dabigatran (Selleck Chemicals, Houston, TX, USA), argatroban (Tokyo Chemical Industry, Tokyo, Japan) and darexaban (Santa Cruz Biotechnology, Santa Cruz, CA, USA).

### 2.2. Cell Lines and Transient Transfection

HEK293FT is an immortalized cell line derived from human fetal kidney. A pair of previously described 293FT-based reporter cell lines that constitutively express individual split reporters (DSP1-7 and DSP8-11 proteins) [9] were used in this study and maintained in Dulbecco’s modified Eagle’s medium (DMEM) containing 10% fetal bovine serum (FBS) and 1 μg/mL puromycin. For establishment of stable cell lines expressing the S protein of SARS-CoV-2, or MERS-CoV, recombinant pseudotype lentiviruses were produced using HEK293T cells with lentiviral transfer plasmid expressing S protein, psPAX2 packaging plasmid and vesicular stomatitis virus (VSV)-G-expressing plasmid. For establishment of stable cell lines expressing ACE2 or CD26, and TMPRSS2, recombinant pseudotype retroviruses expressing one of these proteins were produced using plat-E cells with a VSV-G-expressing plasmid [10]. 293FT-derived reporter cells infected with pseudotype viruses were selected with 1 μg/mL puromycin, 10 μg/mL blasticidin and 300 μg/mL hygromycin for at least 1 week. These bulk selected cells were used to perform fusion assays. Calu-3 cells (ATCC HTB-55) and H3255 cells (CVCL_6831), lung epithelial cell-derived immortalized cells established from human lung cancer, were used as target cells for the fusion and viral infection assays. For establishment of ACE2-knockout Calu-3 cells, lentiviruses were produced by transfecting lentiCRISPRv2 vector (#52961 Addgene, Watertown, MA, USA) with the following gRNA sequences. The gRNA sequences used were 5′-GCT TTC ACG GAG GTT CGA CG and 5′-ATG TTG CAG TTC GGC TCG AT for control, and 5′-ATG AGC ACC ATC TAC AGT AC and 5′-TGC TGC TCA GTC CAC CAT TG for ACE2-knockout. Pooled Calu-3 cells infected with pseudotype viruses were selected with 1 μg/mL puromycin. 

### 2.3. Construction of Expression Vectors

Expression vectors for CD26 and TMPRSS2 were described previously [8]. A synthetic DNA corresponding to the S gene of SARS-CoV-2 (NC_045512.2) was generated by Taihe Gene (Beijing, China). For construction of expression vectors for ACE2, the ACE2 gene was cloned into a pMXs-internal ribosome entry site (IRES)-blasticidin retroviral vector [10]. For construction of expression vectors for S protein of SARS-CoV-2 and MERS-CoV, the coding regions were cloned into a lentiviral transfer plasmid (CD500B-1, SBI, Palo Alto, CA, USA).

### 2.4. DSP Assay to Monitor Membrane Fusion

For the DSP assay using 293FT cells, effector cells expressing S protein with DSP8-11 and target cells expressing CD26 or ACE2, and TMPRSS2 with DSP1-7 were seeded in 12-well cell culture plates (2 × 10^5^ cells/500 μL) one day before the assay. Two hours before the DSP assay, cells were treated with 6 μM EnduRen (Promega, Madison, WI, USA), a substrate for Renilla luciferase, to activate EnduRen. One microliter of each protease inhibitor or anticoagulant dissolved in dimethyl sulfoxide (DMSO) was added to the 384-well plates (Greiner Bioscience, Frickenhausen, Germany). Next, 50 μL of each single cell suspension (effector and target cells) was added to the wells using a Multidrop dispenser (Thermo Scientific, Waltham, MA, USA). After incubation at 37 °C for 4 h, the RL activity was measured using a Centro xS960 luminometer (Berthold, Germany). For the DSP assay using Calu-3 or H3255 cells, target cells were seeded in 384-well plates (2 × 10^4^ cells/50 μL) one day before the assay. Two hours before the DSP assay, cells were treated with 6 μM EnduRen. One microliter of each protease inhibitor or anticoagulant dissolved in DMSO was added to the 384-well plates with 9 μL of culture medium. Next, 40 μL of single cell suspension (effector cells) was added to the wells using a Multidrop dispenser.

### 2.5. Western Blotting

Western blot analysis was performed as described previously [11]. The primary antibodies used were rabbit anti-ACE2 (1:1000, ab15348 Abcam, Cambridge, MA, USA) and anti-GAPDH (1:1000, sc-25778 Santa Cruz Biotechnology, Dallas, TX, USA). HRP-linked donkey anti-rabbit IgG (NA934, GE Healthcare, Piscataway, NJ, USA).

### 2.6. Isolation of SARS-CoV-2

VeroE6 (ATCC CRL-1586) cells were maintained in Eagle’s minimal essential media (MEM) containing 10% FBS. The cells were incubated at 37 °C with 5% CO_2_, and regularly tested for mycoplasma contamination by using PCR and were confirmed to be mycoplasma-free. Respiratory swabs were obtained from a patient with laboratory-confirmed COVID-19, who was hospitalized at the Center Hospital of the National Center for Global Health and Medicine, Tokyo, Japan. The swabs were submitted to the Division of Virology, Department of Microbiology and Immunology, Institute of Medical Science, the University of Tokyo for virus isolation by inoculating with VeroE6 cells. The research protocol was approved by the Research Ethics Review Committee of the Institute of Medical Science of the University of Tokyo. SARS-CoV-2 viruses were propagated in VeroE6 cells in Opti-MEM I (Invitrogen) containing 0.3% bovine serum albumin (BSA) and 1 µg of L-1-Tosylamide-2-phenylethyl chloromethyl ketone (TPCK)-trypsin/ml at 37 °C. All experiments with SARS-CoV-2 viruses were performed in enhanced biosafety level 3 (BSL3) containment laboratories at the University of Tokyo, which are approved for such use by the Ministry of Agriculture, Forestry and Fisheries, Japan.

### 2.7. SARS-CoV-2 Infection Assay

Calu-3 (ATCC HTB-55) cells were maintained in MEM containing 10% FBS. VeroE6/TMPRSS2 [12] (JCRB 1819) cells were propagated in the presence of 1 mg/ml geneticin (G418; Invivogen) and 5 μg/mL plasmocin prophylactic (Invivogen) in DMEM containing 10% FBS. All cells were incubated at 37 °C with 5% CO2, and regularly tested for mycoplasma contamination by PCR and were confirmed to be mycoplasma-free. Using VeroE6/TMPRSS2 or Calu-3 cells as target cells, in vitro infection experiments were composed of two groups, “pretreatment” and “no-pretreatment”. In the “pretreatment” group, cells were pretreated with nafamostat mesylate (10-fold serial dilutions from 100 μM to 1 nM, 4 wells for each dose) for 1 h before infection. SARS-CoV-2 was then added at a multiplicity of infection (MOI) of 0.01 or 0.1 in the absence of TPCK-trypsin and cells were further incubated for 30 min for viral entry. The culture medium was then changed to fresh medium with the same concentrations of nafamostat mesylate as those before infection. In the “no-pretreatment” group, cells were incubated with fresh medium for 1 h in the absence of nafamostat mesylate before infection. SARS-CoV-2 was then added and further incubated for 30 min. The culture medium was then changed to fresh medium containing nafamostat mesylate as in the “pretreatment” group. Cells untreated with nafamostat mesylate before and after infection were also included as a control. Three (for VeroE6/TMPRSS2) or 5 (for Calu-3) days after infection, EC_50_ was determined using the Spearman–Karber formula [13] based on the appearance of visually detectable cytopathic effect (CPE) in quadruplicate experiments.

## 3. Results

**Figure 1:**
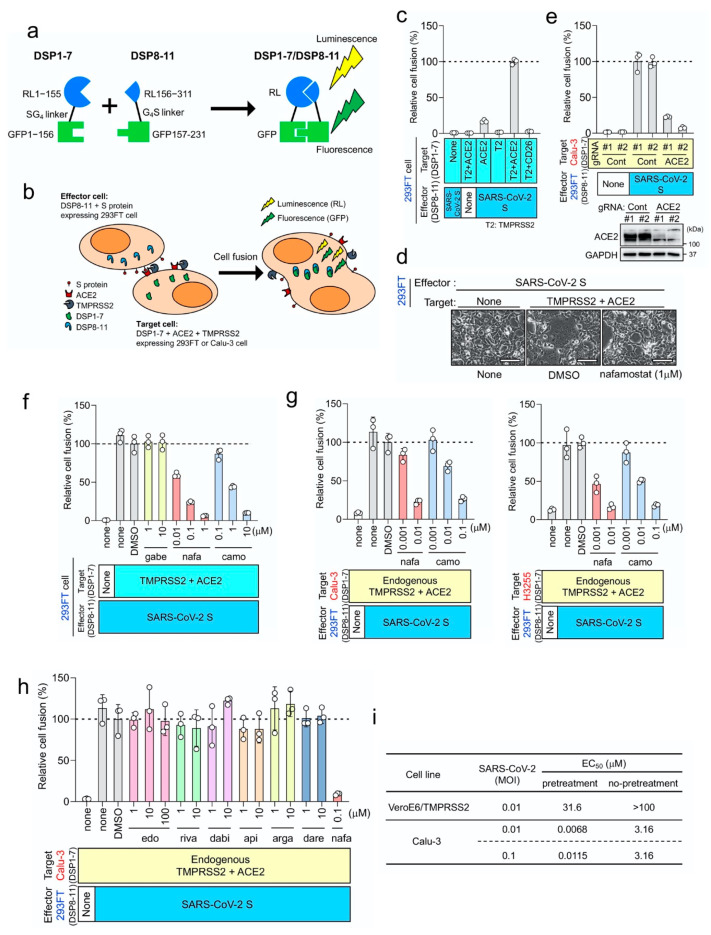
Nafamostat mesylate potently inhibits transmembrane serine protease 2 (TMPRSS2)-dependent severe acute respiratory syndrome coronavirus 2 (SARS-CoV-2) entry into lung epithelium-derived Calu-3 cells. (**a**) Dual split protein (DSP)1-7 has the structure Renilla luciferase (RL)1–155-Ser-Gly-Gly-Gly-Gly-green fluorescent protein (GFP)1–156. DSP8-11 has the structure Met-RL156–311-Gly-Gly-Gly-Gly-Ser-GFP157–231. DSP1-7 and DSP8-11 reassociate efficiently, resulting in reconstitution of functional RL and GFP to generate luminescent and fluorescent signals, respectively. (**b**) Effector cells (293FT cells expressing DSP8-11 and S protein) and target cells (293FT or Calu-3 cells expressing DSP1-7, angiotensin I converting enzyme 2 (ACE2) and TMPRSS2) were co-cultured. Both GFP (fluorescence) and RL (luminescence) signals were generated following DSP1-7 and DSP8-11 reassociation upon cell fusion. (**c**) Different combinations of the effector and target cells were cocultured, and the resulting RL activity was measured. Relative cell-fusion values were calculated by normalizing the RL activity of each co-culture to that of the co-culture of cells expressing S protein with those expressing both receptor and TMPRSS2, which was set to 100%. (**d**) Phase contrast images of SARS-CoV-2 S protein-mediated-cell fusion. Scale bars, 100 μm. (**e**) The fusion assay using wild type and ACE2-kockout Calu-3 cells. (**f**) Three clinically used pancreatitis and/or anticoagulant drugs were evaluated by the DSP assay for their effects on SARS-CoV-2 S-mediated membrane fusion. Relative cell-fusion value was calculated by normalizing the RL activity for each co-culture to that of the co-culture with dimethyl sulfoxide (DMSO) alone, which was set to 100%. gabe: gabexate mesylate, nafa: nafamostat mesylate, camo: camostat mesylate. (**g**) The DSP assay using Calu-3 (left) or H3255 (right) cells as target cells. DSP1-7 was constitutively expressed in Calu-3 and H3255 cells. (**h**) The DSP assay using Calu-3 cells was performed in the presence of various anticoagulants: edo, edoxaban; riva, rivaroxaban; dabi, dabigatran; api, apixaban; arga, argatroban; dare, darexaban. (**i**) In the “pretreatment” group, cells were pretreated with nafamostat mesylate (10-fold serial dilutions from 100 μM to 1 nM, 4 wells for each dose) for 1 h before infection. SARS-CoV-2 was then added and further incubated for 30 min. The culture medium was then changed to fresh medium with the same concentrations of nafamostat mesylate as those before infection. In the “no-pretreatment” group, cells were incubated with fresh medium for 1 h without nafamostat mesylate. SARS-CoV-2 was then added and further incubated for 30 min. The culture medium was then changed to fresh medium containing nafamostat mesylate as in the “pretreatment” group. Three (VeroE6/TMPRSS2) or 5 (Calu-3) days after infection, effective concentration (EC)_50_ was determined using the Spearman–Karber formula [13] based on the appearance of visually detectable cytopathic effect (CPE) in quadruplicate experiments.

Based on our previous work [8] together with others’ [14], we established an experimental system monitoring SARS-CoV-2 S protein-mediated membrane fusion using DSP reporter (Figure 1a), and tested ACE2 and TMPRSS2 dependence in 293FT cells, which do not express ACE2 and TMPRSS2 (Figure 1b–d). Efficient fusion occurred only when both ACE2 and TMPRSS2 were expressed in the target cells, and lack of ACE2 or TMPRSS2 resulted in a significant reduction in fusion (Figure 1c), indicating that the cell fusion largely depends on ACE2 and TMPRSS2 as previously reported for MERS-CoV S protein-mediated fusion, which depends on CD26 and TMPRSS2 (Supplementary Materials, Figure S1a). Interestingly, ACE2 alone induced fusion at a low but detectable level (Figure 1c) while CD26 alone scarcely induced fusion mediated by MERS-CoV S protein (Supplementary Materials, Figure S1a). Although the TMPRSS2-independent component in the SARS-CoV-2 S protein-mediated fusion was significantly lower than the TMPRSS2-dependent component, the result may suggest the presence of some undiscovered mechanism of SARS-CoV-2 S protein-mediated fusion. Furthermore, when ACE2-kockout Calu-3 cells, endogenously expressing ACE2 and TMPRSS2, were used as target cells, fusion scarcely occurred, suggesting that no other functional receptor exists in some types of lung epithelial cells (Figure 1e).

We then tested the activity of three existing Japanese drugs (nafamostat mesylate and gabexate mesylate used for pancreatitis and DIC, and camostat mesylate used for pancreatitis and reflux esophagitis) with inhibitory activity against serine proteases to inhibit SARS-CoV-2 S protein-mediated fusion (Figure 1f). Luciferase activities derived from cells carrying the preformed DSP1-7/DSP8-11 reporter complex were not affected by the drugs (Supplementary Materials, Figure S1b), indicating that the suppression of luciferase activities reflects the inhibition of fusion by drugs. Nafamostat mesylate showed greatest activity, camostat mesylate was 10-fold less active, and gabexate mesylate was inactive within the range of concentrations tested (10 nM to 10 μM; Figure 1d,f), which mirrors drug sensitivity profiles to MERS-CoV S protein-mediated fusion [4] (Supplementary Materials, Figure S1c).

We next used lung epithelium-derived Calu-3 and H3255 cells as target cells, because they endogenously express ACE2 and TMPRSS2, and might better reflect physiological conditions than 293FT kidney cells ectopically expressing ACE2 and TMPRSS2. Nafamostat mesylate inhibited SARS-CoV-2 S protein-mediated membrane fusion in the range of 1–10 nM while camostat mesylate required 10–100 nM to achieve a similar extent of inhibition (Figure 1g). A similar inhibition profile was observed when MERS-CoV S protein-mediated fusion was analyzed [4] (Supplementary Materials, Figure S1d). Therefore, nafamostat mesylate was 10-fold more potent than camostat mesylate. Furthermore, drug concentrations required for fusion inhibition in lung epithelial cells were 10-fold lower than those required for inhibition using 293FT cells (Figure 1f,g).

Various computational approaches have recently been applied to find existing medications targeting TMPRSS2 [15,16]. Consistent with our results, these studies ranked nafamostat mesylate significantly higher than camostat mesylate. It should be noted that several worldwide commercially available anticoagulants, which are serine protease inhibitors targeting Factor Xa or Thrombin, are listed. Therefore, we checked whether these anticoagulants affect SARS-CoV-2 S protein-mediated membrane fusion. Contrary to expectations, they all failed to inhibit membrane fusion (Figure 1h).

Our DSP assays identified nafamostat mesylate as a potent inhibitor of SARS-CoV-2 S-mediated membrane fusion (Figure 1f,g). This inhibition was most likely a result of inhibition of TMPRSS2 on the plasma membrane [2]. SARS-CoV-2 infects Calu-3 cells primarily via the TMPRSS2-dependent plasma membrane pathway [14], while it infects VeroE6/TMPRSS2 cells via the TMPRSS2-independent and cathepsin-dependent endosome pathway in addition to the plasma membrane pathway [17]. We then proceeded to evaluate the effects of nafamostat mesylate on actual SARS-CoV-2 infection in both types of cells. The in vitro infection experiments were performed with or without pretreatment with nafamostat mesylate before infection, as described in Material and Methods as the “pretreatment” or “no-pretreatment” group, respectively. In both groups, nafamostat mesylate was included in culture medium after infection. Based on the appearance of cytopathic effect (CPE), EC_50_ values for Calu-3 cells were 6.8–11.5 nM (pretreatment) and 3.16 μM (no-pretreatment) while those for VeroE6/TMPRSS2 cells were 31.6 μM (pretreatment) and >100 μM (no-pretreatment; Figure 1i). In both cell types, EC_50_ values were much smaller when cells were pretreated with nafamostat mesylate, implying that nafamostat mesylate inhibits TMPRSS2-dependent SARS-CoV-2 entry.

## 4. Discussion

Our findings clearly indicate that nafamostat mesylate, the most effective TMPRSS2 inhibitor so far reported, potently inhibits SARS-CoV-2 S protein-mediated fusion in a cell fusion assay system and viral infection in vitro in a cell-type-dependent manner. Furthermore, EC_50_ values for Calu-3 cells with the pretreatment were around 10 nM, similar to our previous findings with MERS-CoV infection [8]. This extremely high sensitivity to nafamostat mesylate may be because the TMPRSS2-dependent entry pathway predominates in lung epithelium-derived Calu-3 cells [8,14,18]. Camostat mesylate-mediated inhibition of TMPRSS2 significantly reduced SARS-CoV-2 infection of primary human airway epithelial cells [14], suggesting that the TMPRSS2-dependent entry pathway is likely dominant in lung epithelial cells. This idea is also supported by the single-cell RNA-seq data analysis demonstrating high expression of TMPRSS2 in alveolar type 1 and 2 cells [5,6]. Given that blood concentrations of nafamostat mesylate were maintained at 30–240 nM when it was administered intravenously through continuous infusion according to the standard protocol for DIC patients [19], nafamostat mesylate could be used to treat COVID-19. In contrast, significantly higher doses of nafamostat mesylate are required to block SARS-CoV-2 infection of monkey kidney VeroE6/TMPRSS2 cells as reported previously [20], which is probably due to the significant contribution of the TMPRSS2-independent, cathepsin-dependent endosome pathway [17]. Since a pharmacokinetics study using rats revealed the maximum concentration of intact nafamostat mesylate in the lung after infusion to be about 60-fold higher in comparison with the maximum blood concentration [21], such an accumulation may partially suppress SARS-CoV-2 infection of VeroE6/TMPRSS2-like cells, in which the endosome entry pathway predominates [17]. Therefore, identification and characterization of cells that play a key role in virus spread and disease development are required.

Hoffmann et al. have recently reported that nafamostat mesylate blocks activation of SARS-CoV-2 [22]. However here we clearly demonstrated that nafamostat mesylate blocked the membrane fusion step of the virus entry and its activity to block SARS-CoV-2 infection was cell type dependent. These findings are crucial for developing therapeutic strategies. It has been reported recently that abnormal coagulation with elevated concentrations of D-dimer, characteristic of DIC with enhanced fibrinolysis, may influence the prognosis of COVID-19 [23,24]. Furthermore, in a murine asthma model, nafamostat mesylate attenuates respiratory inflammation by blocking activation of NF-κB, a critical transcription factor for inflammatory cytokine production [25]. Therefore, nafamostat mesylate is expected to have multiple therapeutic effects. Since nafamostat mesylate has been prescribed in Japan for many years and adequate clinical data regarding safety have accumulated, we suggest that it should be evaluated in COVID-19 patients by itself or in combination with other antiviral drugs that target separate processes needed for virus production. 

## Supplementary Materials

The following are available online at https://www.mdpi.com/1999-4915/12/6/629/s1, Figure S1: Nafamostat mesylate potently inhibits the TMPRSS2- and CD26-dependent MERS-CoV S protein-mediated membrane fusion.
